# Identification of Novel Genes and Biological Pathways That Overlap in Infectious and Nonallergic Diseases of the Upper and Lower Airways Using Network Analyses

**DOI:** 10.3389/fgene.2019.01352

**Published:** 2020-01-17

**Authors:** Erin E. Baschal, Eric D. Larson, Tori C. Bootpetch Roberts, Shivani Pathak, Gretchen Frank, Elyse Handley, Jordyn Dinwiddie, Molly Moloney, Patricia J. Yoon, Samuel P. Gubbels, Melissa A. Scholes, Stephen P. Cass, Herman A. Jenkins, Daniel N. Frank, Ivana V. Yang, David A. Schwartz, Vijay R. Ramakrishnan, Regie Lyn P. Santos-Cortez

**Affiliations:** ^1^ Department of Otolaryngology, School of Medicine, University of Colorado Anschutz Medical Campus, Aurora, CO, United States; ^2^ Department of Pediatric Otolaryngology, Children's Hospital Colorado, Aurora, CO, United States; ^3^ Department of Medicine, School of Medicine, University of Colorado Anschutz Medical Campus, Aurora, CO, United States

**Keywords:** cholesteatoma, immune pathways, lower airway, mucosa, networks, otitis media, RNA-sequencing, sinusitis

## Abstract

Previous genetic studies on susceptibility to otitis media and airway infections have focused on immune pathways acting within the local mucosal epithelium, and outside of allergic rhinitis and asthma, limited studies exist on the overlaps at the gene, pathway or network level between the upper and lower airways. In this report, we compared [1] pathways identified from network analysis using genes derived from published genome-wide family-based and association studies for otitis media, sinusitis, and lung phenotypes, to [2] pathways identified using differentially expressed genes from RNA-sequence data from lower airway, sinus, and middle ear tissues, in particular cholesteatoma tissue compared to middle ear mucosa. For otitis media, a large number of genes (*n* = 1,806) were identified as differentially expressed between cholesteatoma and middle ear mucosa, which in turn led to the identification of 68 pathways that are enriched in cholesteatoma. Two differentially expressed genes *CR1* and *SAA1* overlap in middle ear, sinus, and lower airway samples and are potentially novel genes for otitis media susceptibility. In addition, 56 genes were differentially expressed in both tissues from the middle ear and either sinus or lower airways. Pathways that are common in upper and lower airway diseases, whether from published DNA studies or from our RNA-sequencing analyses, include chromatin organization/remodeling, endocytosis, immune system process, protein folding, and viral process. Taken together, our findings from genetic susceptibility and differential tissue expression studies support the hypothesis that the unified airway theory wherein the upper and lower respiratory tracts act as an integrated unit also applies to infectious and nonallergic airway epithelial disease. Our results may be used as reference for identification of genes or pathways that are relevant to upper and lower airways, whether common across sites, or unique to each disease.

## Introduction

The unified airway theory proposes that the respiratory tract acts as an integrated unit, from the middle ear through the distant bronchioles (Krouse, 2008). Structurally, the mucosae of the middle ear, nose/sinuses, and lower respiratory tract are highly similar, lined by mostly ciliated epithelium, which is involved in the transport of mucosa and particulate matter. Additionally, bacterial communities in healthy lungs are highly similar to those in the upper respiratory tract (Charlson et al., 2011; Segata et al., 2012; Hanshew et al., 2017). The unified airway model has typically been applied to allergic rhinitis and asthma, with the observations that allergic rhinitis is present in at least 80% of asthma patients, and that asthma is found in up to 40% of patients with allergic rhinitis (Feng et al., 2012; Giavina-Bianchi et al., 2016). In addition, treatment of allergic rhinitis symptoms has been found to improve asthma symptoms and pulmonary function. This is believed to be due to a shared inflammation model, with local inflammatory processes producing systemic mediators that affect disease in other areas of the respiratory tract (Krouse, 2008). Specifically, it has been found that if one area of the airway mucosa is stimulated with antigen, within hours system-wide inflammatory changes are observed. Additionally, atopic patients undergoing surgery for otitis media (OM) with effusion have similar cellular and cytokine profiles in both the middle ear effusion and nasopharynx (Nguyen et al., 2004). It is hypothesized that the middle ear is capable of participating in a T_H_2 inflammatory response and that the inflammation in OM with effusion is not limited to the middle ear (Nguyen et al., 2004).

Limited nonallergic observations of the unified airway have been described in the literature. At least 40% and up to 88% of chronic obstructive pulmonary disease (COPD) patients have sinonasal symptoms, which are increased during COPD exacerbations (Hens et al., 2008; Burgel, 2015). In addition, a study of sinus CT in bronchiectasis patients found that the severity of sinus disease was worse in bronchiectasis patients than in allergic rhinitis patients (Ramakrishnan et al., 2013). In our study, we wanted to further apply the unified airway theory to infectious and nonallergic airway epithelial disease in the middle ear, sinus, and lung. We predicted that the host genetic background contributes to susceptibility to upper and lower airway epithelial diseases, with the hypothesis that genes and enriched pathways identified from either DNA or RNA studies will be shared between upper and lower airway diseases.

## Methods

The study is divided into three parts (**Supplementary Methods**; **Supplementary Figure 1**): ***Part 1*** consists of network analyses and pathway identification using published genes based on genome-wide significant variants from DNA studies and eGenes derived from expression quantitative trait loci (eQTL); ***Part 2*** includes analyses of RNA-sequence data from middle ear, sinus, and lung tissue, and identification of common genes and pathways from network analyses across different sites; and ***Part 3*** involves the comparison of network analysis results from DNA literature and RNA-sequence data in order to find common genes and pathways across the upper and lower airways.

### Network Analyses for Published Genes and eGenes (Part 1)

#### Generation of Gene Lists From the Literature and UK Biobank

A literature search on DNA studies was performed for upper and lower airway phenotypes, including OM, chronic rhinosinusitis (CRS) and/or nasal polyps (NP), chronic bronchitis, bronchiolitis, acute bronchitis, pneumonia, pulmonary nontuberculous mycobacterial (NTM) infection, pulmonary tuberculosis (PTB), and bronchiectasis. Specific terms used for the search and the exclusion criteria are listed in the **Supplementary Methods**. Only studies with genome-wide significant results were included. Genome-wide significance criteria were as follows: [1] variant or gene identified using linkage analyses in family-based studies (LOD≥3.3); [2] variant or gene identified by population-based genome-wide association study (GWAS; *p* < 5.0x10^-8^ if using single-variant analyses, *p* < 2.5x10^-6^ if using gene-based tests). The variants and genes meeting these criteria are included in **Supplementary Table 1** that lists the design, sample size and ancestry of each cited study cohort from which the power of each study may be assessed. Aside from published literature, genome-wide significant variants (*p* < 5.0x10^-8^) were extracted from publicly available GWAS results on selected phenotypes from the UK Biobank [**Supplementary Table 2**, (Neale, 2018)]. Variants classified as “low confidence” in the UK Biobank data set were removed from further analyses.

From variants identified by single-variant GWAS either in literature or the UK Biobank, only the most significant variant in each peak was selected for further analyses and annotated using the hg19 version of the UCSC Variant Annotation Integrator (Kent et al., 2002; Hinrichs et al., 2016). Furthermore, variants were annotated as eQTLs using the GTEx v7 portal (GTEx Portal, 2017). In this study, we identified the significant eGenes for the variants identified from the literature or UK Biobank in 26 selected tissues (**Supplementary Table 3**) and annotated the results using Ensembl BioMart (Zerbino et al., 2018). Note that GTEx has eQTL data for lung but not for middle ear or sinonasal tissues, thus we were limited to identification of eQTLs based on other mucosal, respiratory or lymphoid tissues in GTEx (**Supplementary Table 3**). Multiple significant eGenes were typically identified for each variant, but intergenic variants that were not identified as eQTLs for the 26 tissues selected in GTEx were not considered further.

Gene lists were compiled for each phenotype (**Supplementary Table 4**), which includes the following: [1] Genes were significant by gene-based GWAS or linkage analyses from the literature. [2] From the literature and UK Biobank, for variants identified by single-variant GWAS, genes were only included if the variant was located in a gene (coding, intronic, or UTR), not if it was upstream or downstream. [3] For all types of variants from single-variant GWAS whether from literature or the UK Biobank, eGenes were identified from single-tissue eQTL analysis in GTEx. Duplicate genes were removed within each list. Additionally, the genes identified for the lower airway phenotypes were combined into a single list (“Lower”).

#### Network Analysis for Lists of Published Genes and eGenes

NetworkAnalyst was used to generate networks (Xia et al., 2014; Xia et al., 2015; Zhou et al., 2019). The input used were the gene lists identified from the literature and UK Biobank for Part 1 (**Supplementary Table 4**), with separate networks created for OM, CRS, and Lower (**Supplementary Methods**). Networks were created using the Generic PPI, with the IMEx Interactome database from InnateDB (Breuer et al., 2013). The default network creation method was used for the module and PANTHER Biological Process (BP) analyses, which adds in the first neighbors (interacting genes) for the seed genes (genes on the input list). Module analysis was performed on each subnetwork, to break the larger subnetworks into smaller, more densely connected clusters or modules (Xia et al., 2014), using the Walktrap algorithm (Pons and Latapy, 2005). Nodes representing genes within a module are likely to work collectively to perform a biological function. When phenotypes were combined, a combined network was created in NetworkAnalyst and visualized using Cytoscape software, in order to delineate overlaps and differences between phenotypes (Shannon et al., 2003; Assenov et al., 2008; Doncheva et al., 2012).

PANTHER BP enrichment analysis was completed for each significant module within the larger subnetworks (Mi et al., 2019). Each node (gene) is annotated with PANTHER BP Gene Ontology (GO) Terms or pathways. PANTHER uses a subset of GO Terms to simplify and condense results. The output of the PANTHER BP enrichment analysis are the pathways that are enriched in the nodes in the module or subnetwork. Significant pathways [false-discovery rate (FDR)-adjusted *p* < 0.05] were compiled into a final list for each phenotype (**Supplementary Table 5**). The Multiple List Comparator (http://www.molbiotools.com/listcompare.html) was used to make comparisons and generate Venn diagrams for either gene or pathway lists.

### Network Analyses Using RNA-Sequence Data for Upper and Lower Airway Phenotypes (Part 2)

#### RNA-Sequencing for Middle Ear Tissues From Individuals With OM

Prior to start of the study, recruitment of patients undergoing OM surgery was approved by the Colorado Multiple Institutional Review Board. All study participants provided written informed consent. Three cholesteatoma samples (considered “case” tissue) and four middle ear mucosa samples (“control” tissue) were collected from patients undergoing OM surgery at the University of Colorado Hospital or Children's Hospital Colorado, and these samples were submitted for RNA-sequencing (RNA-Seq). For cholesteatoma samples, the median RIN was 5.8 and median DV% was 89.2, while for mucosa samples median RIN was 1.5 and median DV% was 52.8. Tissue samples were processed as described in the **Supplementary Methods**. Libraries were constructed using the NuGEN Trio RNA-Seq kit (Tecan, Redwood City, CA, USA), which includes an rRNA depletion step. Sequencing was completed on the Illumina NovaSeq, with paired-end 2x151bp reads. An average of 11.3 million read pairs were obtained per sample (range 4.5 to 23.2 million read pairs). One sample (3086) was removed from further analyses due to an insufficient mapping rate to the human genome (5%) and not clustering with the other OM samples in the principal components analysis (**Supplementary Figure 2**).

#### RNA-Seq Data for CRS, NTM, and COPD

Previously, uncinate mucosa tissue from three patients with CRS and four control individuals underwent RNA-Seq (Ramakrishnan et al., 2017). For lung phenotypes, a search of the NCBI Gene Expression Omnibus (GEO) database (http://www.ncbi.nlm.nih.gov/geo/) did not identify transcriptome data on lower airway tissue biopsies. However two RNA-Seq data sets were available on bronchoalveolar lavage (BAL) fluid samples for NTM cases and controls [GSE103852, unpublished, three cases and three controls] and large airway brushings for COPD cases and controls [GSE124180, (Morrow et al., 2019), three COPD cases and four controls, all without emphysema]. Raw RNA-Seq results were not available for the COPD data set, and therefore we used the nonnormalized count data that was available. On the other hand, the CRS and NTM data sets had the raw read data available for analysis.

#### Processing of RNA-Seq Data and Differential Expression Analysis

Reads were trimmed with either Trimmomatic for the CRS and NTM data sets or BBDuk software for OM (Bolger et al., 2014; Bushnell et al., 2017). Transcripts were quantified using Salmon, run in mapping-based mode, which includes indexing and quantification (Patro et al., 2017). The tximport package was used to extract counts from the salmon quantification output (Soneson et al., 2015). The DESeq2 workflow was followed for the tximport steps and DESeq2 analyses [http://bioconductor.org/packages/devel/bioc/vignettes/DESeq2/inst/doc/DESeq2.html, (Love et al., 2014)].

For OM, CRS, and NTM, the nonnormalized counts from tximport were used for the DESeq2 analyses. For COPD, the nonnormalized counts were available in the GEO database and were used as the input. Counts were filtered to have an average of more than three reads in either the cases or controls. The plotPCA function in DESeq2 was used to generate principal components (PC) plots for each data set. DESeq2 was run with default parameters, and included read count normalization followed by differential expression analysis. DESeq2 analysis was performed for each of the four phenotypes individually (OM, CRS, NTM, COPD). Multiple testing correction was performed using adjustment for FDR, with significance threshold for differentially expressed genes (DEGs) at adj-*p* < 0.05.

#### Network Analysis for Differentially Expressed Genes

For Part 2, network analysis using the same workflow as in section 2.1.2 was performed using NetworkAnalyst with the DEGs and fold-change as input. A “Lower” list was created that combined the DEGs for NTM and for COPD, while OM and CRS were analyzed separately. Chord and Venn diagrams were created to compare the DEGs across OM, CRS, and Lower phenotypes. Significant pathways were compiled into a final list for each phenotype group (**Supplementary Table 5**). Venn diagrams were also made to quantify pathway overlaps among phenotypes.

#### Literature Review for Transcriptome Studies

A literature search on RNA studies was performed for upper and lower airway phenotypes, using the same workflow as in section 2.1.1. Specific terms used for the search are included in the **Supplementary Methods**. Studies were excluded if RNA was not extracted from the disease tissue of interest. Genome-wide significance was not required for transcriptome studies. Articles meeting the criteria were summarized (**Supplementary Table 6**).

### Comparisons Between Published and RNA-Seq Data (Part 3)

In order to detect concordance between genome-wide significant genes and eGenes (Part 1) and DEGs from RNA-Seq data (Part 2), the gene lists from each part were compared by phenotype (OM, CRS, Lower) and Venn diagrams were created. Likewise, comparisons were made between Parts 1 and 2 for lists of pathways by phenotype.

## Results

### Genes and Pathways Identified in the Literature and UK Biobank GWAS (Part 1)

Upon review of the literature, 46 genome-wide significant variants and 64 genes were identified from GWAS and family-based studies (**Supplementary Table 1**). No variants overlap between phenotypes, but there is some overlap at the gene level, namely, *HLA-DRB1* in both OM and pneumonia and *MUC6* in both bronchiolitis and pneumonia. In addition, 40 significant variants and 21 genes from the Neale UK Biobank single-variant GWAS were identified (**Supplementary Table 2**). Two variants (rs338598 and rs34210653 in CRS/NP) and three genes (*ALOX15*, *CYP2S1*, and *FOXP1* in CRS/NP) were identified in both the literature and UK Biobank data. The literature source for the CRS/NP data was a paper that performed a GWAS in both deCODE and UK Biobank, so the results would be expected to overlap (Kristjansson et al., 2019). When only the most significant variant in each peak was selected from single-variant GWAS, 84 genome-wide significant variants remained and were queried in the GTEx portal for association with gene expression levels (eQTLs). In total, 122 eGenes were identified from 84 variants (**Supplementary Table 3**). Eighteen variants were excluded because the variant was not located in a gene and was not a significant eQTL in the selected GTEx tissues. **Table 1** lists the final gene counts for each phenotype, including OM and CRS. The lower airway phenotypes (bronchiolitis, chronic bronchitis, pneumonia, and acute bronchitis, and NTM and PTB) were included in a single “Lower” list for further analyses. Fifteen genes (8%) overlap between upper and lower airway phenotypes (**Table 1**; **Supplementary Table 4**). The majority of these genes are immune-related (**Supplementary Table 4**).

**Table 1:** Counts of published genes and eGenes by phenotype (Part 1)^1^.

Phenotype	Published genes^2^	eGenes^3^	Total genes	References
OM	19	49	68	(Allen et al., 2013; Santos-Cortez et al., 2015; Einarsdottir et al., 2016; van Ingen et al., 2016; Tian et al., 2017; Santos-Cortez et al., 2018)
CRS	16	25	41	(Kristjansson et al., 2019)
Bronchiolitis	17	30	47	(Salas et al., 2017)
Chronic Bronchitis	4	1	5	(Lee et al., 2014; Cho et al., 2015; Dijkstra et al., 2015)
Pneumonia and Acute Bronchitis	18	21	39	(Kenyan Bacteraemia Study Group et al., 2016; Hayden et al., 2017; Tian et al., 2017; Salas et al., 2018)
NTM and PTB	9	12	21	(Curtis et al., 2015; Sveinbjornsson et al., 2016; Chen et al., 2017; Zheng et al., 2018)
All phenotypes combined	81	99	180	–
Overlap between upper and lower airways	1	14	15 (8%)	–

^1^Genes are listed in **Supplementary Table 4**.

^2^Published genes include genes that harbor genome-wide significant variants.

^3^eGenes were identified in eQTL analyses using published genome-wide significant variants that control expression in 26 selected tissues in the GTEx database.

In the network created from the combined gene list for OM, CRS, and Lower (**Supplementary Figure 3**), there is minimal overlap between the upper and lower airways at a gene level. The different phenotypes are interconnected in the network, but the phenotypic associations are not necessarily with the same genes.

In order to generate lists of significant pathways for each phenotype, we also analyzed the networks per phenotype. The network input was the gene list for each phenotype (OM, CRS, and Lower), and the network was created based on known protein-protein interactions. For OM, four subnetworks were generated, composed of 20 modules, all of which were significant. For CRS, four subnetworks were generated, composed of 10 modules, all of which were significant. For Lower, two subnetworks were generated, composed of 22 modules, 21 of which were significant. PANTHER BP enrichment analysis was completed on each module individually. Significantly enriched GO Terms/pathways were identified and compiled into a single list for each phenotype (**Supplementary Table 5**). Significant pathways were identified for OM (*n* = 36), CRS (*n* = 13), and Lower (*n* = 37; **Supplementary Figure 4**). Overall, based on published genes and eGenes, 22 pathways (41%) overlap between the upper and lower airways, and seven pathways were common to all three phenotypes (OM, CRS, and Lower, **Supplementary Figure 4**). These seven pathways include antigen processing and presentation, endocytosis, immune system process and response, protein folding, and viral process (**Supplementary Figure 4**).

### Genes and Pathways Identified by RNA-Seq (Part 2)

**Figure 1:**
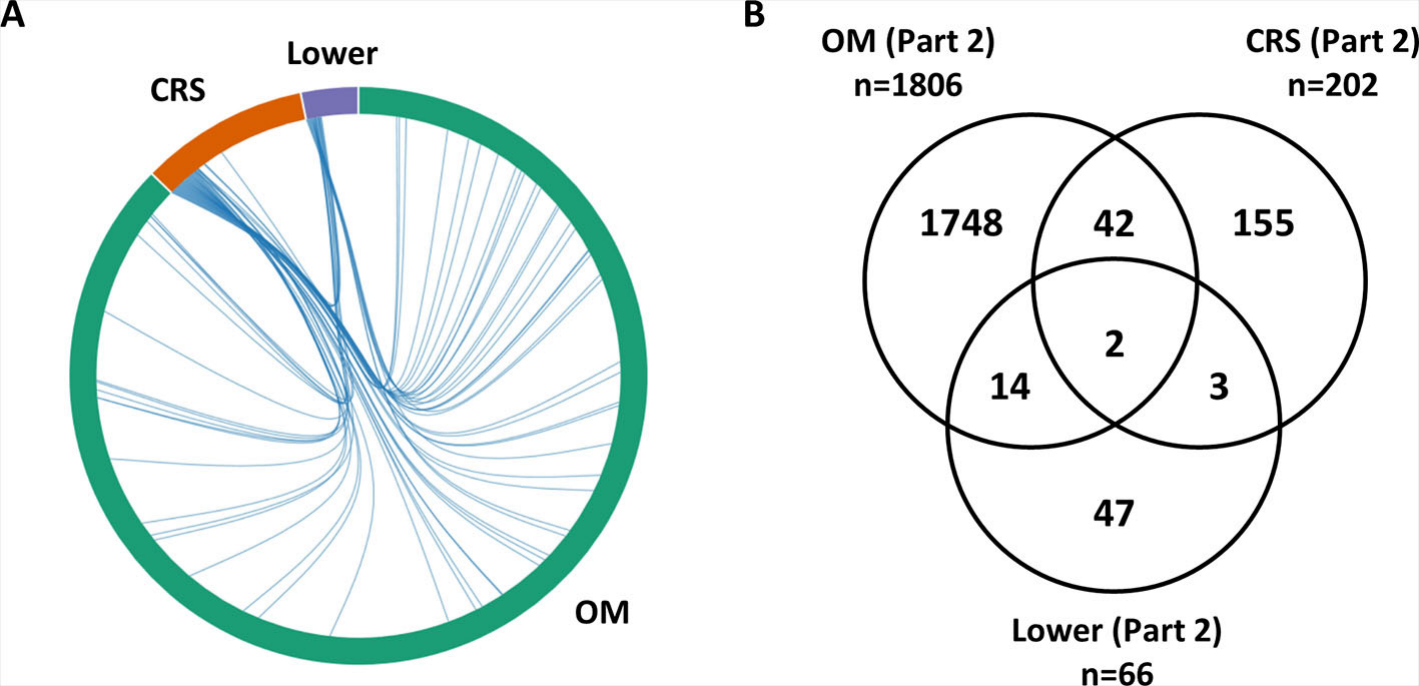
Overlap between lists of differentially expressed genes from RNA-Seq data (Part 2). **(A)** The chord diagram presents the overlap between the differentially expressed genes (DEGs) for each phenotype (otitis media [OM], chronic rhinosinusitis [CRS], Lower including nontuberculous mycobacterial [NTM and chronic obstructive pulmonary disease [COPD]). The connecting lines within the chord diagram show the overlap, at a gene level, between phenotypes. **(B)** Venn diagram showing the overlap in the Part 2 gene lists between OM, CRS, and Lower (**Supplementary Table 6**). Nineteen genes (0.9%) overlap between upper and lower airway phenotypes.

Differential expression analysis was completed separately for each phenotype (OM, CRS, Lower). For OM, a large number of genes (*n* = 1,806) were identified as differentially expressed between cholesteatoma and middle ear mucosa (**Supplementary Table 7**). Overall, 19 genes (0.9%) overlap between upper and lower airway phenotypes (**Figure 1**). The two DEGs that are present in all three phenotypes are *CR1* and *SAA1* (**Supplementary Table 7**). Three DEGs were shared between CRS and Lower, namely, *RDH10, SAA2*, and *SLC7A11* (**Supplementary Table 7**). Of the 14 DEGs that were identified in OM and Lower data sets, half of the genes are known to perform various enzymatic functions, while four genes are involved in gene regulation (**Supplementary Table 7**).

From networks identified in OM RNA-Seq data, four subnetworks were generated, composed of 96 modules, 16 of which were significant. For CRS, six subnetworks were generated, composed of 64 modules, 56 of which were significant. For Lower, three subnetworks were generated, composed of 29 modules, 26 of which were significant. Using the same PANTHER BP enrichment analysis workflow in Part 1 and the DEGs as input, significant pathways were identified for OM (*n* = 68), CRS (*n* = 56), and Lower (*n* = 34; **Supplementary Figure 5**; **Supplementary Table 5**). Overall, based on DEGs, 32 pathways (38%) overlap between the upper and lower airways, and 27 pathways were common to all three phenotypes (OM, CRS, and Lower; **Supplementary Figure 5**; **Supplementary Table 5**). Notably about half of these 27 pathways that were common in OM, CRS, and Lower also overlap with DEGs identified in previous microarray and RNA-Seq studies (Liu et al., 2004; Kwon et al., 2006; Lee et al., 2006; Payne et al., 2008; Raju et al., 2008; Stankovic et al., 2008; Rostkowska-Nadolska et al., 2011; Klenke et al., 2012; Macias et al., 2013; Wang et al., 2016; Ramakrishnan et al., 2017; Wang et al., 2017; Gao et al., 2018a; Gao et al., 2018b; Kato et al., 2018; Langelier et al., 2018; Ninomiya et al., 2018; Okada et al., 2018; Jovanovic et al., 2019; Walter et al., 2019; Yao et al., 2019). The common pathways from Part 2 RNA-Seq data that were identified in the transcriptome literature are apoptosis, cell adhesion, cell cycle, cell proliferation, chromatin organization/remodeling, endocytosis, glycogen metabolic process, immune system process, muscle contraction, protein phosphorylation, proteolysis, RNA metabolic process, and transcription (**Supplementary Table 6**).

### Comparison of Genes and Pathways From the Literature or UK Biobank GWAS vs. RNA-Seq Data (Part 3)

**Figure 2:**
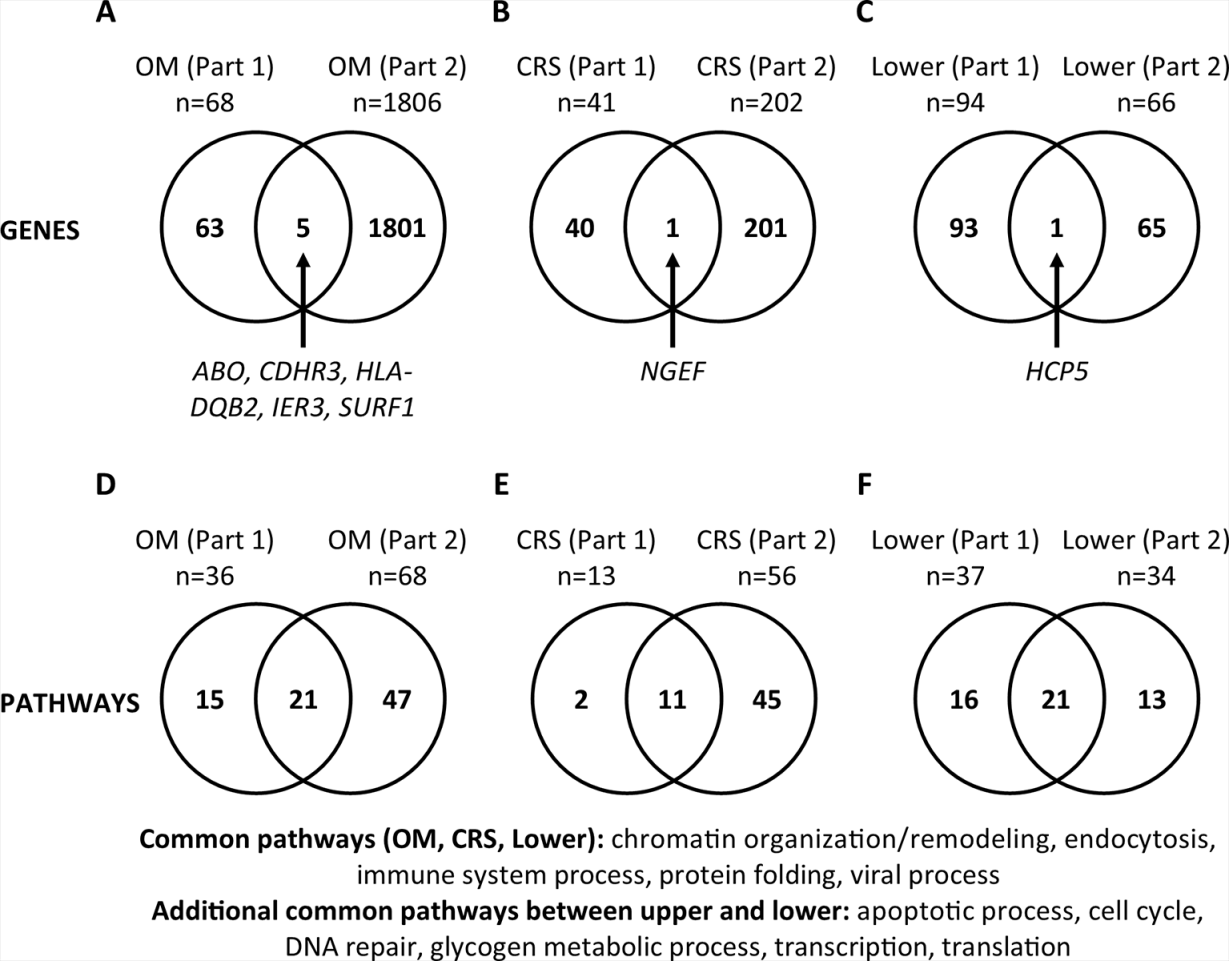
Comparisons between gene and pathway lists (Part 3). **(A**–**C)** Significant genes were compared between Part 1 (published genes and eGenes) and Part 2 (RNA-Seq) for each phenotype (otitis media [OM], chronic rhinosinusitis [CRS], and Lower). Genes are listed in **Supplementary Tables 4** and **6**. **(D**–**F)** The lists of significant PANTHER Biological Process Gene Ontology (GO) Terms/pathways were compared between Parts 1 and 2 for each phenotype (**Supplementary Figures 3** and **5**; **Supplementary Table 5**). Twelve (35%) pathways were present in both the upper and lower airway phenotypes (**Supplementary Table 5**).

For each phenotype (OM, CRS, and Lower), we compared the genes and pathways identified from review of literature on DNA studies vs. RNA-Seq results in order to determine if commonalities in genetic background in the susceptibility to upper and lower airway diseases are supported by both types of studies (**Figure 2**). For OM, five genes (*ABO, CDHR3, HLA-DQB2, IER3, SURF1*, 0.3%) were identified in both the DNA literature (Part 1) and RNA-Seq (Part 2). For CRS, only the *NGEF* gene (0.4%) was identified in both Parts 1 and 2. For the lower airway, the *HCP5* gene (0.6%) was identified in both Parts 1 and 2. Of note, there are a large number of genes that do not overlap between Parts 1 and 2 for all three phenotypes. In addition, all genes common between Parts 1 and 2 were unique to each phenotype.

The same comparisons were made at the pathway level (**Supplementary Table 5**; **Figure 2**). For OM, 21 pathways (25%) were identified in the literature (Part 1) and RNA-Seq (Part 2). For CRS, 11 pathways (19%), and for the lower airway, 21 pathways (42%) were identified in both Parts 1 and 2. We also looked at the pathways that were common between Parts 1 and 2 individually for each phenotype, then compared them across the phenotypes. The common pathways among the three phenotypes (OM, CRS, and Lower) are chromatin organization/remodeling, endocytosis, immune system process, protein folding, and viral process (**Figure 2**). The additional pathways that are common between the upper and lower airway phenotypes are apoptotic process, cell cycle, DNA repair, glycogen metabolic process, transcription, and translation. In total, 12 (35%) pathways overlap between upper and lower airway phenotypes (**Supplementary Table 5**; **Figure 2**).

## Discussion

Here, we report the results of an investigation of the unified airway theory in infectious and nonallergic airway epithelial conditions, both for genetic susceptibility (Part 1) and differentially expressed genes (Part 2). Five pathways namely chromatin organization/remodeling, endocytosis, immune system process, protein folding, and viral process were shown to overlap among OM, CRS, and Lower airway phenotypes in both Part 1 and Part 2 (**Figure 2**), indicating that our approach is a viable route for finding overlaps among significant pathways for diseases across the upper and lower airways. Three of these pathways (chromatin organization/remodeling, endocytosis, and immune system process) were also identified in previously published transcriptome studies (**Supplementary Table 6**). These three pathways have the strongest evidence for involvement in both the upper and lower airway infectious and nonallergic disease processes. While this study was focused on the unified airway theory and therefore the pathways that are common between OM, CRS, and the Lower airway, the pathways that are unique to each disease site are also of interest, particularly those that overlap between DNA and RNA studies (**Supplementary Table 5**). These pathways may also provide insight into disease-specific susceptibility and pathogenesis.

In each Part of this study, we identified more overlap between upper and lower airway at the pathway level than at the gene level (**Figure 1**; **Supplementary Figures 4** and **5**; **Supplementary Table 5**). This finding may indicate that the genes responsible differ between the diseases, but that those genes impact the same pathways and cause disease or disease susceptibility in a similar manner. While Part 1 measures changes that occur at the DNA level and relate to genetic susceptibility for a disease, Part 2 measures changes that occur at the RNA level. The latter may be related to the genetic susceptibility and downstream processes that are affected, or they may be related to the changes that occur as a result of the disease process itself. While we saw overlap in the results from Part 1 and Part 2, especially at the pathway level, it is reasonable to see that the results did not overlap completely. On the other hand, genes and pathways that overlap in both DNA and RNA studies for the same or similar phenotype(s) provide strong evidence for their involvement in disease.

Cholesteatoma is a middle ear lesion of keratinized epithelium surrounding squamous debris that usually occurs as part of chronic OM, and is characterized by uncontrolled growth and proliferation. This is the first report using RNA-Seq in OM patients with middle ear cholesteatoma compared to middle ear mucosal tissue as a control. Previous studies have used either skin or granulation tissue as control tissue in cholesteatoma studies (Kwon et al., 2006; Klenke et al., 2012; Macias et al., 2013; Gao et al., 2018a; Jovanovic et al., 2019). In our study, 1,806 genes were differentially expressed between cholesteatoma and mucosa samples (**Supplementary Table 7**; **Figure 1**). This large number of DEGs may be explained by the growth characteristics of the cholesteatoma tissue. In addition, 68 pathways were enriched in cholesteatoma tissue (**Supplementary Table 5**). These RNA-Seq findings may provide insight into the disease mechanism for cholesteatoma, which is still poorly understood. Both the DEGs and pathways identified here provide a resource for future studies, e.g., for prioritizing candidate genes from sequencing studies whether by DEGs or expression levels in middle ear and sinonasal tissues.

For example, of the five OM genes that overlap between Parts 1 and 2, *CDHR3*, *HLA-DQB2*, and *IER3* are annotated with viral process, apoptotic process, and DNA repair, which are some of the PANTHER BP terms that were found as overlapping pathways in Parts 1 and 2. *HLA-DQB2* (MIM 615161) is a well-known immune gene, while *IER3* (MIM 602996) regulates genes involved in apoptosis. *CDHR3* (MIM 615610) encodes a transmembrane epithelial protein and was previously identified in GWAS for childhood ear infections (Pickrell et al., 2016) and also for asthma exacerbations in children primarily due to viral respiratory infections (Bønnelykke et al., 2014; Everman et al., 2019). While the GWAS finding of *CDHR3* variant rs114947103 as a protective factor against OM has not been replicated (Pickrell et al., 2016), the downregulation of *CDHR3* in cholesteatoma compared to mucosal middle ear tissue (adj-*p* = 0.004) in our RNA-Seq study strongly supports a role for *CDHR3* in OM.

However because *CDHR3* was identified in a GWAS for asthma, this gene was not included in overlaps between OM and Lower airway. Of the 14 DEGs that overlap between OM and Lower, all the genes except *MUCL1* are in overlapping pathways in OM and Lower (**Supplementary Table 5**). On the other hand, there are 42 DEGs that overlap between OM and CRS in the RNA-Seq data, and are not shared with the Lower airway phenotype (**Figure 1**). This suggests that at the gene level there are more genes that overlap between OM and CRS (*n* = 42) compared to OM and Lower airway (*n* = 14) or between the upper and lower airway overall (*n* = 19). The shared number of DEGs between OM and CRS at the RNA-Seq level may help explain why, in addition to the physical proximity and connectedness of the middle ear to the sinonasal complex, OM and CRS are more similar to each other than either is to the Lower airway phenotypes.

By comparing the RNA-Seq results from middle ear, sinus, and lung, we identified two potentially novel genes for OM, i.e. *CR1* and *SAA1*, that are involved in susceptibility to both upper and lower airway disease. *CR1* (MIM 120620), which encodes the complement C3b/C4b receptor 1 (Knops blood group), was a significant DEG in OM, CRS, and Lower (−3.6, +1.8, and −1.5 log2 fold change, respectively). The GO BP pathway annotations for *CR1* include immune system process, viral process, and negative regulation of complement activation. CR1 is important for the host response to bacteria, and mediates immune adherence and phagocytosis (Smith et al., 2002; Li et al., 2010). In CRS, CR1 was reported to have denser localization in the mucosa of CRS patients than in normal mucosa (Miyaguchi et al., 1988), and higher levels of CR1 were found in granulocytes from the circulation and sinus pus in patients with purulent sinusitis (Berg et al., 1989). In pneumonia, CR1 had significantly higher levels on neutrophils in patients with bacterial pneumonia compared to those with viral pneumonia (Hohenthal et al., 2006). The *CR1* gene was reported to be important for host defense against pneumococcal infection in mice (Ren et al., 2004). In addition, deficiency of CR1 was reported in a patient with OM, sinusitis, and pneumonia (Sadallah et al., 1999). In our study, *CR1* was found to be downregulated in OM cholesteatoma and NTM BAL, but upregulated in CRS. This could be consistent with a potential deficiency of CR1 resulting in susceptibility to infections in the OM and NTM patients.

*SAA1* (MIM 104750), encoding serum amyloid A1, was significantly differentially expressed in OM, CRS, and Lower (−2.5, +2.9, −4.6 log2 fold change, respectively). The *SAA1* GO BP annotations include positive regulation of cytokine secretion, receptor-mediated endocytosis, and positive regulation of cell adhesion. Elevated plasma levels of SAA1 is a well-documented clinical indicator for inflammatory conditions, and is suggested to have a role in host defense against bacterial infection. *SAA1* has been reported to be upregulated in lung parenchyma and bronchi of patients with COPD compared with smoking controls (Lopez-Campos et al., 2013). During TB treatment, *SAA1* expression is reduced, and the reduction is greater for patients who culture-converted at later time points (Sigal et al., 2017; Kedia et al., 2018). In our study, this gene was found to be downregulated in OM cholesteatoma and COPD large airway brushings, but upregulated in CRS. Interestingly for both *CR1* and *SAA1* the direction of regulation of expression is opposite in OM vs. CRS but the same in OM vs. Lower. This may be primarily due to the differences in types of tissues used; alternatively it may also be due to disease-specific processes in each site.

One limitation of the OM RNA-Seq study is that we were unable to collect paired cholesteatoma and mucosa samples from the same patients due to technical reasons. This prevents us from comparing the expression levels of genes in the same patients, which could provide additional valuable information about the disease process. Other limitations are [1] the small amount of middle ear tissues available resulting in lower RIN values, and [2] the small sample size for RNA-Seq studies. On the other hand, the rRNA depletion protocol used for RNA-Seq allowed us to have analyzable data for comparison and genetic results were replicated in other data sets, indicating that our main findings are not false-positive results. Nevertheless following up these findings in a well-powered cohort particularly for OM will help validate the identified DEGs, enable identification of additional novel DEGs, and also allow for inclusion of covariates such as patient age, sex and ethnicity. Based on our OM RNA-Seq data set with ~15,000 genes for testing, ~1,800 DEGs, minimum fold change≥2, and average read counts of ~900, we will have sufficient power ≥80% with an expanded data set of 28 middle ear tissues, preferably with paired samples.

In summary, we have confirmed support for the unified airway theory for infectious and nonallergic airway epithelial disease, using both genetic susceptibility and differential tissue expression studies. We also identified two potentially novel genes for OM susceptibility, *CR1* and *SAA1*, in addition to 56 OM DEGs that are also DEGs for CRS or lower airways. Moreover we identified a total of 1,806 DEGs and 68 pathways that are enriched in cholesteatoma compared to middle ear mucosa. In the process we have created a data set that can be used as reference for finding genes or pathways that are relevant to upper and lower airways, whether common across sites, or unique to each disease.

## Data Availability Statement

The data generated for this article can be found in dbGAP, accession phs001941.v1.p1.

## Ethics Statement

The studies involving human participants were reviewed and approved by Colorado Institutional Review Board. Written informed consent to participate in this study was provided by adult participants or for children, the participants' parents.

## Author Contributions

RS-C, VR, DS, IY, and DF conceptualized the study. GF, EH, JD, MM, PY, SG, MS, SC, and HJ collected middle ear samples. TB performed isolation of RNA from tissue samples and submitted RNA samples for sequencing. EB and EL performed RNA sequence analyses. EB and SP extracted data from literature. EB extracted data from UK Biobank and GTEx and performed network analyses. EB and RS-C wrote the manuscript. All authors read and approved the manuscript.

## Funding

This work was funded by the National Institute on Deafness and Other Communication Disorders (NIDCD) of the National Institutes of Health grants R01 DC015004 (to RS-C), K23 DC014747 (to VR), and a training grant T32 DC012280 to the Department of Otolaryngology at the University of Colorado.

## Conflict of Interest

The authors declare that the research was conducted in the absence of any commercial or financial relationships that could be construed as a potential conflict of interest.

## Abbreviations

BAL, bronchoalveolar lavage; COPD, chronic obstructive pulmonary disease; CRS, chronic rhinosinusitis; DEG, differentially expressed gene; eQTL, expression quantitative trait locus; GEO, gene expression omnibus; GO, gene ontology; GTEx, Genotype-Tissue Expression Project; GWAS, genome-wide association study; NP, nasal polyp; NTM, nontuberculous mycobacterial infection; OM, otitis media; PTB, pulmonary tuberculosis; RNA-Seq, RNA-sequencing.
