# Supplementary material for: Identification of Novel Genes and Biological Pathways That Overlap in Infectious and Nonallergic Diseases of the Upper and Lower Airways Using Network Analyses
Source: Front Genet. 2020 Jan 17;10:1352. doi: 10.3389/fgene.2019.01352 (PMC6979043; doi:10.3389/fgene.2019.01352)
Supplement: Supplementary file 1 [file DataSheet_1.zip › SupplementaryFigures.pdf]

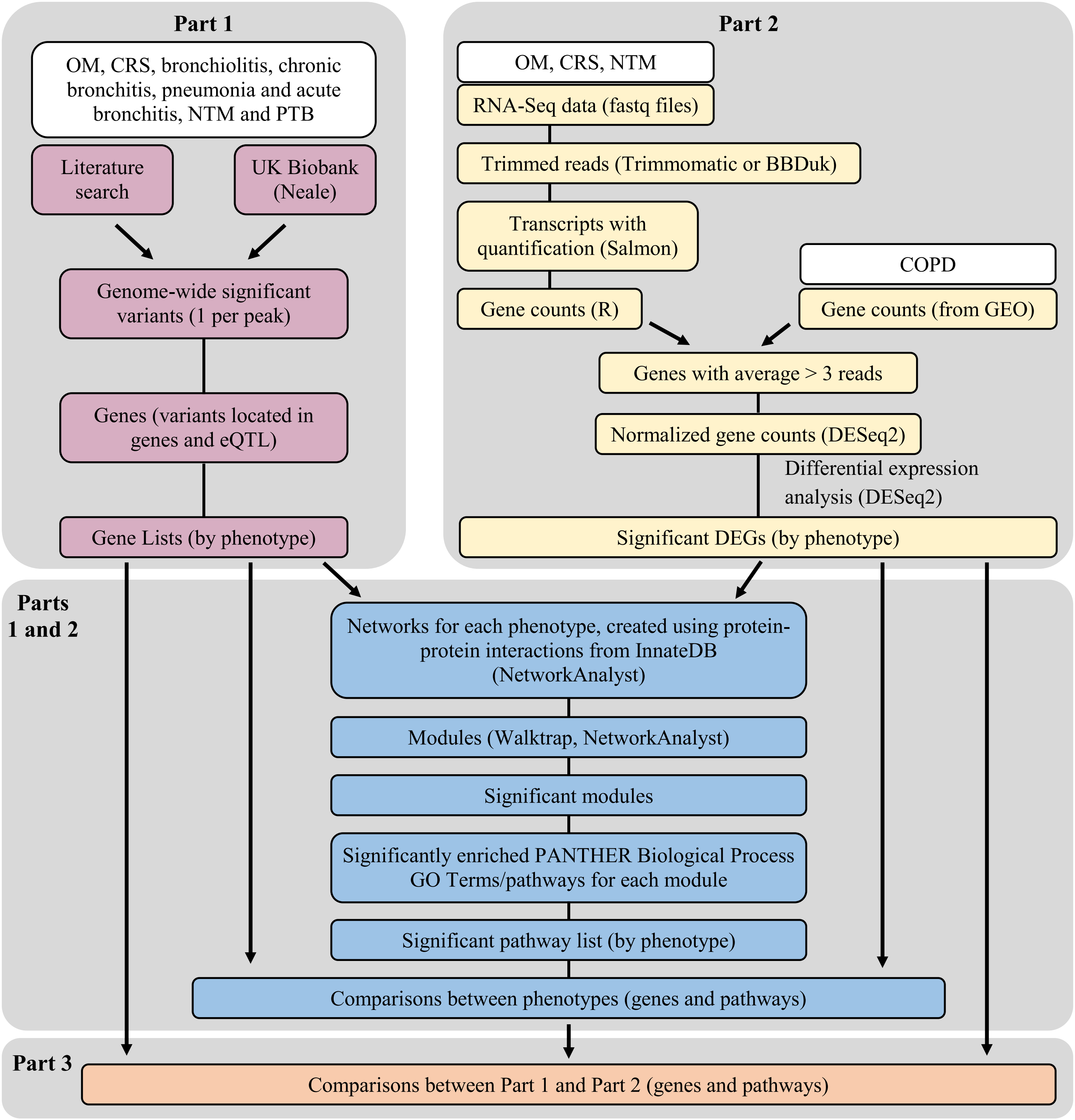


**Supplementary Figure 1: Analysis strategy.**

Legend: *White boxes,* phenotypes; *pink boxes,* steps for Part 1; *yellow boxes,* steps for Part 2; *blue boxes,* steps for both Parts 1 and 2; *peach box,* Part 3. Abbreviations: COPD, chronic obstructive pulmonary disease (non-emphysema); CRS, chronic rhinosinusitis; DEGs, differentially expressed genes; eQTL, expression quantitative trait locus; GEO, gene expression omnibus portal; GO, gene ontology; GWAS, genome-wide association study; NTM, pulmonary nontuberculous mycobacterial infection; OM, otitis media; PTB, pulmonary tuberculosis infection.

A

| ID | sample type | mapping rate |
|---|---|---|
| 3086 | mucosa | 5% |
| 3090 | cholesteatoma | 68% |
| 4006 | mucosa | 32% |
| 4008 | mucosa | 22% |
| 4012 | mucosa | 44% |
| 4013 | cholesteatoma | 64% |
| 4014 | cholesteatoma | 53% |

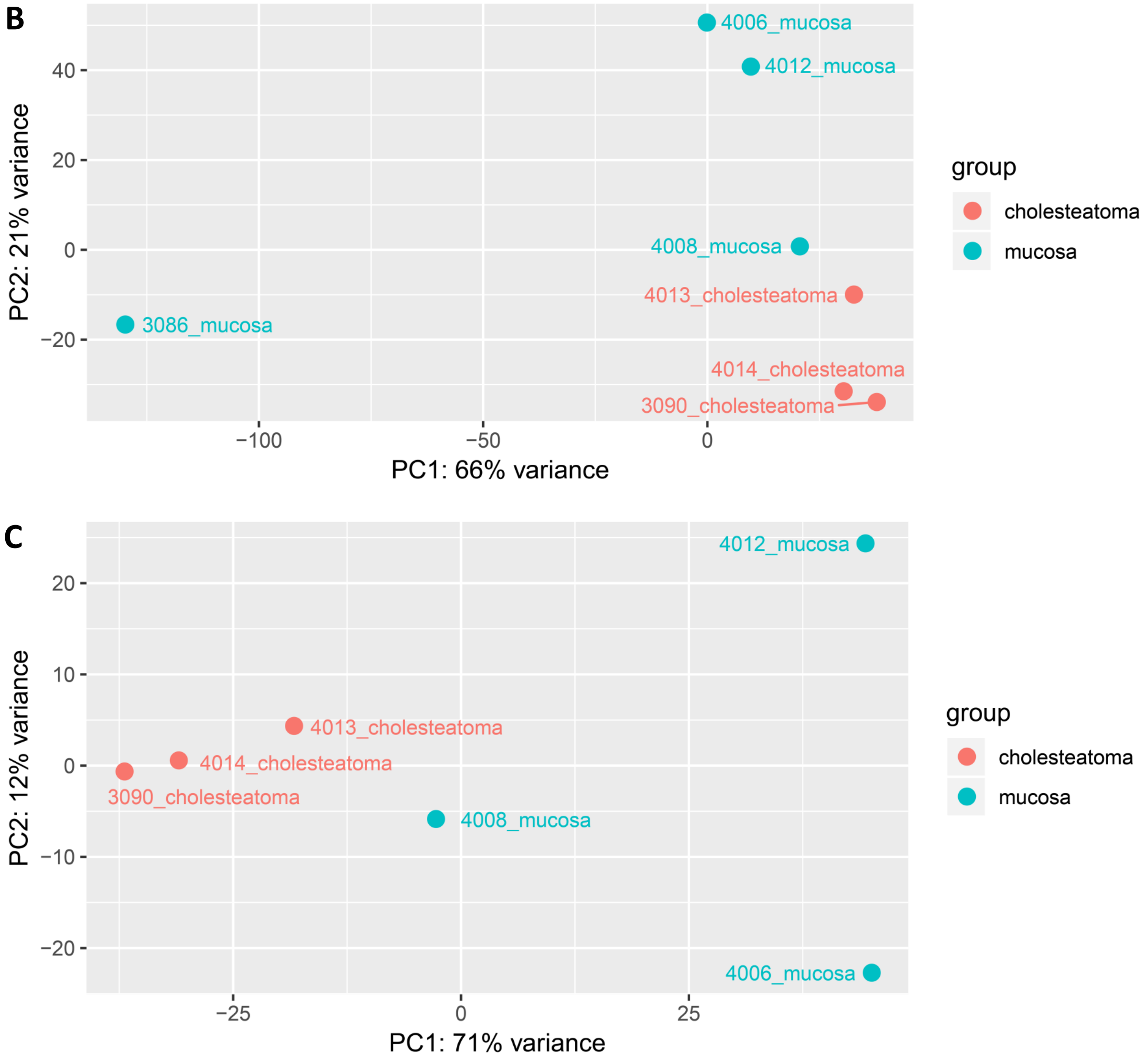


**Supplementary Figure 2. OM RNA-Seq results (Part 2)**

(A) The transcript mapping rate from Salmon for each OM sample. The mucosa sample for 3086 had poor mapping (5%) compared to the other OM tissue samples. (B) Principal components analysis for the OM samples. Note that sample 3086 does not cluster with the other samples. (C) Principal components analysis for OM samples, with the 3086 mucosa sample removed.

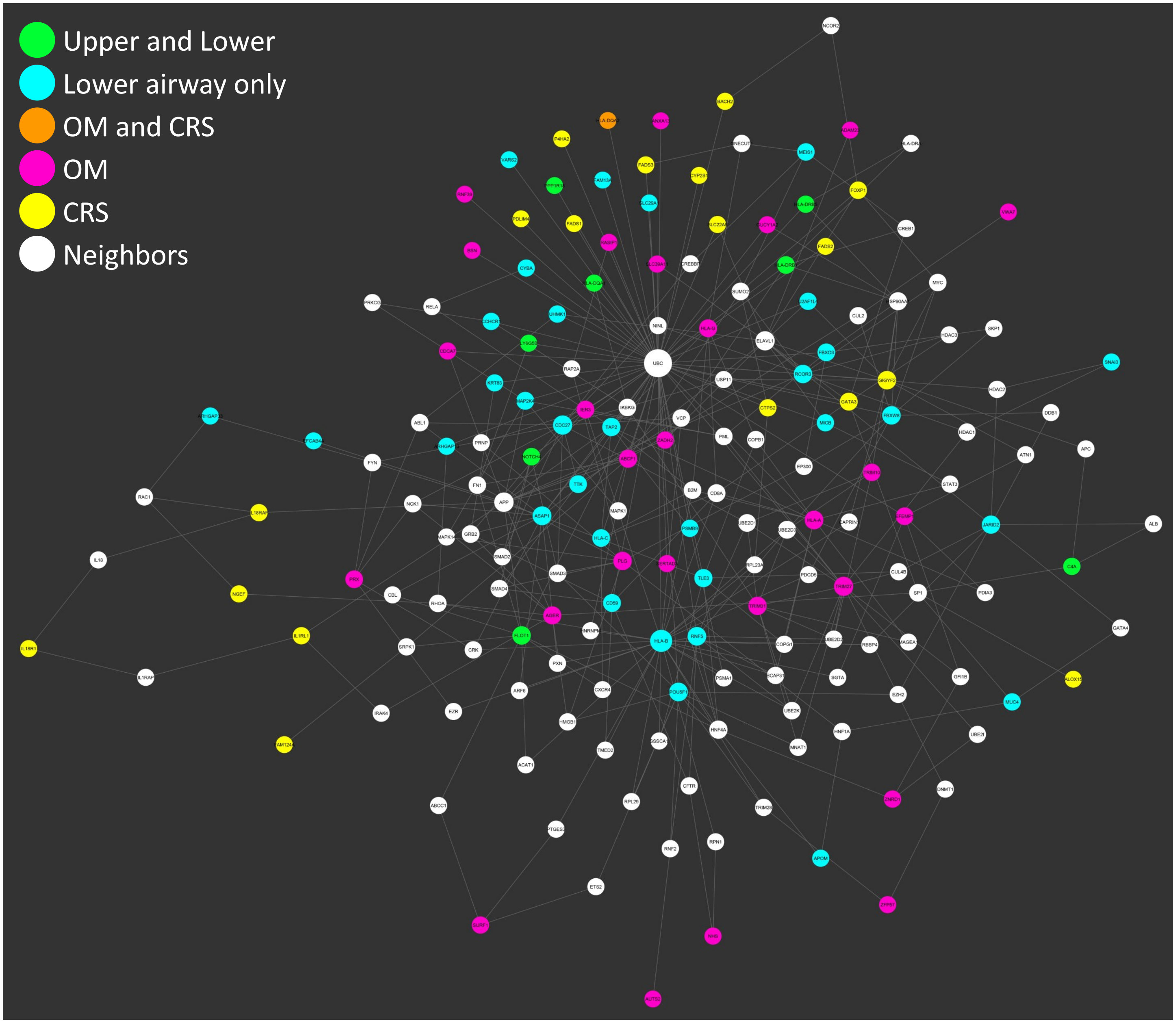


**Supplementary Figure 3. Network image for Part 1, all phenotypes**

Using 180 genes for all phenotypes as input, a minimum network (179 nodes or genes represented as *circles*, 354 edges shown as *lines between nodes*, and 86 seed genes), which includes only the first-neighbor genes that are needed to connect the seed genes, was generated based on known protein-protein interactions from the IMEx Interactome InnateDB database. A second subnetwork was also generated (not shown, 3 nodes, 2 edges, 2 seed genes). The image was generated using Cytoscape software (Shannon et al., 2003; Assenov et al., 2008; Doncheva et al., 2012). The degree of a node is the number of connections it has to other nodes. The size of the nodes in the network is related to the degree, with larger nodes having a higher degree and therefore more connections within the network. Each color represents phenotypes or overlaps between phenotypes, with colors for multiple overlaps overriding single phenotype categories.

A

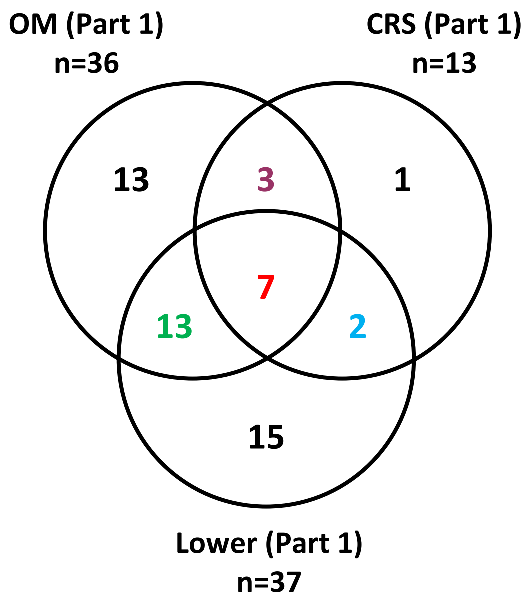


B

| Phenotypes | Pathway |
|---|---|
| OM, CRS, Lower | Antigen processing and presentation |
| OM, CRS, Lower | Endocytosis |
| OM, CRS, Lower | Immune system process and response |
| OM, CRS, Lower | Protein folding |
| OM, CRS, Lower | Viral process |
| OM, Lower | Apoptotic process |
| OM, Lower | Blood coagulation |
| OM, Lower | Cell cycle |
| OM, Lower | Cellular defense response |
| OM, Lower | Chromatin organization |
| OM, Lower | Complement activation |
| OM, Lower | DNA recombination and repair |
| OM, Lower | Endoderm development |
| OM, Lower | Glycogen metabolic process |
| OM, Lower | Protein transport |
| OM, Lower | Regulation of binding |
| OM, Lower | Translation |
| CRS, Lower | Chromatin remodeling |
| CRS, Lower | Transcription by RNA polymerase II |
| OM, CRS | RNA processing and splicing |

**Supplementary Figure 4. Overlapping pathways between OM, CRS, and Lower (Part 1)**

(A) Twenty-two (41%) PANTHER BP pathways overlap between upper and lower airway phenotypes. *Lower* includes the genes identified for bronchiolitis, chronic bronchitis, pneumonia and acute bronchitis, and NTM and PTB. *Red*, OM, CRS and Lower; *green,* OM and Lower; *blue,* CRS and Lower; *purple,* OM and CRS. (B) Overlapping pathways for OM, CRS, and Lower listed in alphabetical order within each category. The ungrouped pathway lists are in Supplementary Table 5.

A

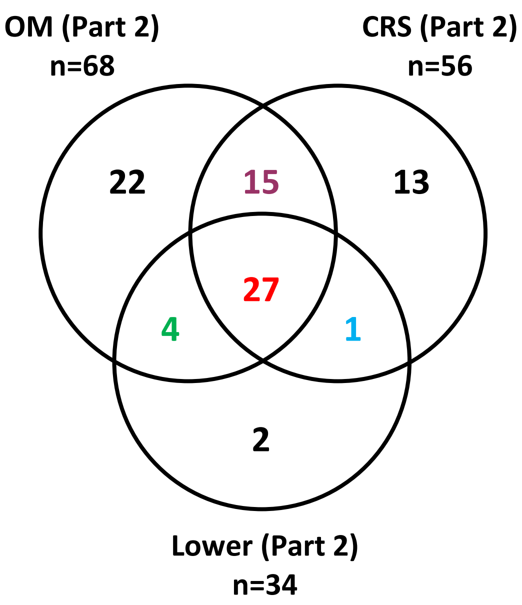


B

| Phenotypes | Pathway |
|---|---|
| OM_CRS_Lower | Apoptotic process (negative regulation) |
| OM_CRS_Lower | Cell adhesion |
| OM_CRS_Lower | Cell cycle |
| OM_CRS_Lower | Cell proliferation |
| OM_CRS_Lower | Chromatin organization/remodeling |
| OM_CRS_Lower | Circadian rhythm |
| OM_CRS_Lower | DNA repair and replication |
| OM_CRS_Lower | Endocytosis |
| OM_CRS_Lower | Immune system process |
| OM_CRS_Lower | Protein acetylation |
| OM_CRS_Lower | Protein folding |
| OM_CRS_Lower | Protein phosphorylation |
| OM_CRS_Lower | Proteolysis |
| OM_CRS_Lower | Regulation of translation |
| OM_CRS_Lower | RNA metabolic process |
| OM_CRS_Lower | RNA splicing and processing |
| OM_CRS_Lower | Transcription |
| OM_CRS_Lower | Viral process |
| OM_Lower | Glycogen metabolic process |
| OM_Lower | MRNA polyadenylation |
| OM_Lower | Muscle contraction |
| OM_Lower | Translation |
| CRS_Lower | DNA recombination |
| OM_CRS | Blood coagulation |
| OM_CRS | Cell differentiation |
| OM_CRS | Cell_matrix adhesion |
| OM_CRS | Cytoskeleton organization |
| OM_CRS | Endoderm development |
| OM_CRS | Heart development |
| OM_CRS | Mitochondrion organization |
| OM_CRS | Phagocytosis |
| OM_CRS | Protein transport |
| OM_CRS | Regulation of binding |
| OM_CRS | Regulation of transcription and transcription factor activity |
| OM_CRS | Response to toxic substance |
| OM_CRS | Skeletal system development |

**Supplementary Figure 5. Overlapping pathways between OM, CRS, and Lower (Part 2)**

(A) Thirty-two (38%) PANTHER BP pathways overlap between upper and lower airway phenotypes. *Lower* includes DEGs for NTM and COPD. *Red*, OM, CRS and Lower; *green,* OM and Lower; *blue,* CRS and Lower; *purple,* OM and CRS. (B) Overlapping pathways for OM, CRS, and Lower listed in alphabetical order within each category. The ungrouped pathway lists are in Supplementary Table 5.
