# Supplementary material for: Identification of Novel Genes and Biological Pathways That Overlap in Infectious and Nonallergic Diseases of the Upper and Lower Airways Using Network Analyses
Source: Front Genet. 2020 Jan 17;10:1352. doi: 10.3389/fgene.2019.01352 (PMC6979043; doi:10.3389/fgene.2019.01352)
Supplement: Supplementary file 1 [file DataSheet_1.zip › SupplementaryMethods.docx]

**Supplementary Methods**

**Literature search (Part 1)**

A literature search on DNA studies was performed in January and February 2019 for upper and lower airway phenotypes, using the disease terms listed below. Specific terms used for the search for each phenotype were “exome,” “genetic linkage,” “GWAS,” “eQTL,” “pQTL” (protein QTL), “genome-wide,” “whole-genome,” “meta-analysis genetic,” “transcriptome,” “microarray,” “RNA-Seq,” and “RNA sequencing.” The genome-wide association study (GWAS) Catalog (<https://www.ebi.ac.uk/gwas/>) was also used to identify potential articles for each phenotype. Only genome-wide genetic studies and genome-wide transcriptome studies were included. All candidate gene studies and articles that were not written in English were excluded. All articles were screened to exclude phenotypes that did not fit the criteria, particularly for chronic bronchitis, for which we only included the airway COPD phenotype, and for NTM/PTB, for which we only included the pulmonary phenotypes. The articles that passed the filtering criteria were organized by type of study (genome or transcriptome) and screened for inclusion in downstream analyses.

Phenotypes included:

# Upper airway

## CRS and/or NP (chronic rhinosinusitis, sinusitis, nasal polyps, nasal polyposis)

## Otitis media (otitis media, cholesteatoma, childhood ear infection)

# Lower airway

## Chronic bronchitis (chronic bronchitis, nonemphysematous, non-emphysematous, airway (NOT asthma, sleep, cystic fibrosis), mucus)

## Bronchiolitis (bronchiolitis)

## Acute Bronchitis (bronchitis)

## Pneumonia (pneumonia, empyema)

## Nontuberculous mycobacterial infection (pulmonary nontuberculous mycobacteria, pulmonary mycobacterium avium)

## Tuberculosis (tuberculosis)

## Bronchiectasis (bronchiectasis)

# Phenotypes excluded:

# Any case reports that seem to be syndromic

# Lung transplant

# Bone marrow transplant-related lung disease

# Cystic fibrosis

# Rheumatoid arthritis-associated lung disease

# TAP/HLA/MHC deficiency-associated lung disease

# Sarcoidosis

# Hypersensitivity (lung disease)

# Agammaglobulinemia

# Immunodeficiency

# Primary ciliary dyskinesia

# Kartagener syndrome

# Mannose binding lectin (MBL) deficiency

For the genome analysis, only studies with genome-wide significant results were included. Genome-wide significance criteria were as follows: [1] gene or variant identified using linkage analyses in family-based studies (LOD≥3.3); [2] variant or gene identified by population-based genome-wide association study (GWAS; *p*<5.0x10^-8^ if using single-variant analyses, *p*<2.5x10^-6^ if using gene-based tests). The variants and genes meeting these criteria are included in Supplementary Table 1.

For the transcriptome analysis, studies were excluded if the RNA was not extracted from the disease tissue of interest (e.g. excluded peripheral blood, cord blood, and cell culture studies). Genome-wide significance was not required for the transcriptome studies. Results from articles meeting the criteria were summarized (Supplementary Table 7).

**UK Biobank GWAS (Part 1)**

Publicly available UK Biobank GWAS results were downloaded for select phenotypes [Supplementary Table 2, (Neale, 2018)]. The GWAS included 361,194 samples (194,174 females and 167,020 males), and included the covariates age, age^2, inferred_sex, age * inferred_sex, age^2 * inferred_sex, and PCs 1-20. Only genome-wide significant variants (p < 5.0x10^-8^) were selected, and variants classified as “low confidence” were removed from further analyses.

**Variant selection (Part 1)**

From both literature and UK Biobank variants identified by single-variant GWAS, only the most significant variant in each peak was selected for further analyses, where a peak was defined by adjacent significant variants within 5 Mb of each other. Variants were annotated with chromosome, genomic position, gene, and variant type from the UCSC Variant Annotation Integrator (hg19), using annotations from UCSC Genes (RefSeq, GenBank, CCDS, Rfam, tRNAs & Comparative Genomics) and dbSNP 151 (Kent et al., 2002; Hinrichs et al., 2016). Variants were only annotated with a gene if they were located in a gene (coding, intronic, or UTR), not if they were upstream or downstream.

**eQTL determination (Part 1)**

Variants were annotated as expression quantitative trait loci (eQTLs) using the GTEx v7 portal data downloaded on 02/21/19 (GTEx Portal, 2017). GTEx has calculated cis-eQTLs for variants located within 1 Mb of the transcription start site for each gene, where the variant alleles alter the expression levels of the corresponding eGenes in that tissue. GTEx calculated a nominal p-value threshold for every gene, and variant-gene pairs below that threshold were considered to be significant. In this study, we identified the significant eGenes for the variants identified from the literature or UK Biobank in 26 selected tissues (Supplementary Table 3), and annotated the results using Ensembl BioMart [GRCh37 release 96, (Zerbino et al., 2018)]. Multiple significant eGenes were typically identified for each variant, but intergenic variants that were not identified as eQTLs for the 26 tissues selected in GTEx were not considered further.

**Gene lists (Part 1)**

Gene lists were compiled for each phenotype (Supplementary Table 4), which includes the following: [1] Genes were significant by gene-based GWAS or linkage analyses from the literature. [2] From the literature and UK Biobank, for variants identified by single-variant GWAS, genes were only included if the variant was located in a gene (coding, intronic, or UTR), not if it was upstream or downstream. [3] For all types of variants from single-variant GWAS whether from literature or the UK Biobank, eGenes were identified from single-tissue eQTL analysis in GTEx. Duplicate genes were removed within each list. Additionally, the genes identified for the lower airway phenotypes were combined into a single list (“Lower”).

**Network analysis for lists of published genes and eGenes (Part 1)**

# NetworkAnalyst was used to generate networks (<https://www.networkanalyst.ca>) (Xia et al., 2014; Xia et al., 2015; Zhou et al., 2019). The input used were the gene lists identified from the literature and UK Biobank for Part 1 (Supplementary Table 4), with separate networks created for OM, CRS, and Lower. Networks were created using the Generic PPI, with the literature-curated IMEx Interactome database from InnateDB (Breuer et al., 2013). After network creation, the subnetworks were displayed, typically with one large subnetwork (“continent”) and several small subnetworks (“islands”). The default network creation method was used for the module and PANTHER Biological Process (BP) analyses, which adds in the first neighbors (interacting genes) for the seed genes (genes on the input list). In the images, nodes within a network are designated with circles, and represent genes (either seed genes or first neighbor genes). Edges are the lines connecting the nodes, and represent published protein-protein interactions.

# Module analysis was performed on each subnetwork, to break the larger subnetworks into smaller, more densely connected clusters or modules (Xia et al., 2014), using the Walktrap algorithm (Pons and Latapy, 2005; Xia et al., 2015). The Walktrap algorithm is based on random walks, with the premise that the walks are more likely to stay within a module, as there are few edges that lead outside the module. Initially, each node is assigned to separate modules. As the Walktrap algorithm runs random walks, it merges the separate modules together, resulting in a final list of modules with associated p-values. The Wilcoxon rank-sum evaluates the difference between the number of edges within a module and the number of edges connecting the nodes of a module with the rest of the network. Only significant modules [false-discovery rate (FDR)-adjusted *p*<0.05] were included in downstream PANTHER BP analyses.

# When phenotypes were combined, a combined network was created in NetworkAnalyst and visualized using Cytoscape software, in order to delineate overlaps and differences between phenotypes (Shannon et al., 2003; Assenov et al., 2008; Doncheva et al., 2012).

# PANTHER BP enrichment analysis was completed for each significant module within the larger subnetworks, and also for each smaller subnetwork that was not divided into modules (Mi et al., 2019). Each node (gene) is annotated with PANTHER BP Gene Ontology (GO) Terms or pathways. PANTHER uses a subset of GO Terms to simplify and condense results. The output of the PANTHER BP enrichment analysis are the pathways that are enriched in the nodes in the module or subnetwork. The FDR-adjusted *p*-value reported for each pathway is useful for comparison of pathways within a module and for the identification of significant pathways, but is not comparable across modules and subnetworks. Significant pathways (FDR-adjusted *p*<0.05) were compiled into a final list for each phenotype (Supplementary Table 5). Duplicate pathways within a phenotype were removed. The PANTHER BP GO-Term annotation set was selected for these analyses as it is a simplified set of annotations that specify the biological systems to which each protein contributes. The Multiple List Comparator (<http://www.molbiotools.com/listcompare.html>) was used to make comparisons and generate Venn diagrams for either gene or pathway lists.

**RNA-Seq for tissues from individuals with OM (Part 2)**

# Study subjects

# Prior to start of the study, recruitment of patients undergoing OM surgery was approved by the Colorado Multiple Institutional Review Board. All study participants gave written informed consent in accordance with the Declaration of Helsinki. A total of eight cholesteatoma (considered “case” tissue) and eight middle ear mucosa samples (“control” tissue) were collected from patients undergoing OM surgery at the University of Colorado Hospital (eight patients) and Children’s Hospital Colorado (four patients). Initially, paired samples were available from four otitis media patients, however only three unpaired cholesteatoma samples and four unpaired mucosa samples passed QC and were submitted for RNA-sequencing (RNA-Seq). For cholesteatoma samples, the median RIN was 5.8 and median DV% was 89.2, while for mucosa samples median RIN was 1.5 and median DV% was 52.8. The ethnicity for individual 3090 was Hispanic, while the ethnicities for the other individuals were non-Hispanic White.

# Tissue processing and RNA extraction

# Tissue samples were collected in the operating room, immediately added to an Oragene RNA kit, and transported to the laboratory on ice. Samples were immediately isolated using the QIAGEN RNeasy Micro Kit and following the supplied protocol for isolation of total RNA from tissue. The tissue was initially homogenized with glass beads and QIAGEN buffer RLT, using a bead vortexer for 4 minutes. The standard protocol was followed after the homogenization step.

# RNA-Seq

# In total, seven tissue samples underwent RNA-Seq (three cholesteatoma and four mucosa samples). Libraries were constructed using the NuGEN Trio RNA-Seq kit (Tecan, Redwood City, CA, USA), which includes an rRNA depletion step. Sequencing was completed on the Illumina NovaSeq, with 40 million reads per sample and paired-end 2x151bp reads. One sample (3086) was removed from further analyses due to an insufficient mapping rate to the human genome (5%) and not clustering with the other OM samples in the principal components analysis (Supplementary Figure 4).

**RNA-Seq data for CRS, NTM, and COPD (Part 2)**

# CRS

# Previously, uncinate mucosa tissue from three patients with CRS and four control individuals underwent RNA-Seq (Ramakrishnan et al., 2017). The ethnicity for Individual ctrl_30 was Hispanic, while the ethnicities for the other individuals were non-Hispanic White. The raw fastq data was re-analyzed using the pipeline described below.

# NTM and COPD

# For lung phenotypes, a search of the NCBI Gene Expression Omnibus (GEO) database (<http://www.ncbi.nlm.nih.gov/geo/>) did not identify transcriptome data on lower airway tissue biopsies. However, two RNA-Seq datasets were available on bronchoalveolar lavage (BAL) fluid (NTM) and large airway brushings (COPD).

# The NTM dataset (GSE103852, unpublished) included RNA-Seq for BAL fluid samples from three individuals with NTM (case) and three individuals without NTM (control), and all individuals were of non-Hispanic White ethnicity. The raw fastq data for the NTM study was available for analysis.

# The COPD study (GSE124180) collected three sets of samples for each individual [BAL, large airway brushings, and peripheral blood, (Morrow et al., 2019)]. Only the large airway brushings data was used for this study. The COPD study included details about the extent of emphysema measured in each patient, as measured by percent emphysema, which is based on image analysis of chest CT data as the percentage of lung voxels below -950 HU. As our project was designed to investigate lower airway disease, only individuals with no emphysema were included (pctemph < 5). The control individuals selected for this analysis were former smokers. All of the case individuals were of non-Hispanic White ethnicity, so four African-American control individuals were excluded from the analysis. There were three case individuals (S1, S8, S16) and four control individuals (S2, S4, S14, S17) that met these criteria. Raw RNA-Seq results were not available for the COPD dataset, and therefore we used the non-normalized count data that was available.

# **Processing of RNA-Seq data (OM, CRS, NTM, Part 2)**

Reads were trimmed with either Trimmomatic (CRS and NTM) or BBDuk (OM) software. Trimmomatic v0.39 was used in single end mode, removed Illumina adapters (2:30:10), removed leading and trailing bases below a threshold quality (LEADING:3, TRAILING:3), used a sliding window trimming approach (SLIDINGWINDOW:4:15), and dropped reads below a minimum length of 36 (MINLEN:36) (Bolger et al., 2014). BBDuk (<https://sourceforge.net/projects/bbmap/>) v.38.50 was used in paired-end mode, removed adapters, and trimmed reads using k-mers (forcetrimleft=1, ktrim=r, k=23, mink=11, hdist=1, tpe=t, tbo=t, minlength=36) (Bushnell et al., 2017).

# Transcripts were quantified using Salmon v0.13.1 (Patro et al., 2017). Salmon was run in mapping-based mode, which includes indexing and quantification. The Salmon index was created using the Ensembl GRCh38 human reference genome (homo_sapiens.GRCh38.cdna.all.fa.gz) with a k-mer setting of 31. The ‘--validateMappings’ flag was used for transcript quantification.

# The tximport package in R was used to extract counts from the salmon quantification output (Soneson et al., 2015). The DESeq2 workflow (<http://bioconductor.org/packages/devel/bioc/vignettes/DESeq2/inst/doc/DESeq2.html>) was followed for the tximport steps and DESeq2 analyses. Briefly, tximport was used to import the salmon output. This resulted in non-normalized counts for each gene.

# **Differential expression analysis (OM, CRS, NTM, COPD) (Part 2)**

# For OM, CRS, and NTM, the non-normalized counts from tximport were used for the DESeq2 analyses. For COPD, the non-normalized counts were available in the GEO database and were used as the input. Counts were filtered to have an average of more than 3 reads in either the cases or controls.

# DESeq2 (Love et al., 2014) was used to generate principal components (PC) plots for each dataset. DESeq2 analysis also included read count normalization and differential expression (DE) analysis. Read count normalization accounts for the sequencing depth and RNA composition of each sample. DE analysis was performed for each of the four phenotypes individually (OM, CRS, NTM, COPD), with a Cook’s cutoff filtering for outliers. Multiple testing correction was performed using adjustment for FDR, with significance threshold for differentially expressed genes (DEGs) at adj-*p*<0.05.

**Network analysis for differentially expressed genes (Part 2)**

For Part 2, network analysis using the same workflow as described for Part 1 was performed using NetworkAnalyst with the DEGs as input. A “Lower” list was created that combined the DEGs for NTM and for COPD, while OM and CRS were analyzed separately. Chord and Venn diagrams were created to compare the DEGs across OM, CRS and Lower phenotypes. Significant pathways were compiled into a final list for each phenotype group (Supplementary Table 5). Venn diagrams were also made to quantify pathway overlaps among phenotypes.

## **Comparisons (Part 3)**

In order to detect concordance between genome-wide significant genes and eGenes (Part 1) and DEGs from RNA-Seq data (Part 2), the gene lists from each Part were compared by phenotype (OM, CRS, Lower) and Venn diagrams were created. Likewise, comparisons were made between Parts 1 and 2 for lists of pathways by phenotype.

# **References**

Assenov, Y., Ramirez, F., Schelhorn, S.E., Lengauer, T., and Albrecht, M. (2008). Computing topological parameters of biological networks. *Bioinformatics* 24(2)**,** 282-284. doi: 10.1093/bioinformatics/btm554.

Bolger, A.M., Lohse, M., and Usadel, B. (2014). Trimmomatic: a flexible trimmer for Illumina sequence data. *Bioinformatics* 30(15)**,** 2114-2120. doi: 10.1093/bioinformatics/btu170.

Breuer, K., Foroushani, A.K., Laird, M.R., Chen, C., Sribnaia, A., Lo, R., et al. (2013). InnateDB: systems biology of innate immunity and beyond--recent updates and continuing curation. *Nucleic Acids Res* 41(Database issue)**,** D1228-1233. doi: 10.1093/nar/gks1147.

Bushnell, B., Rood, J., and Singer, E. (2017). BBMerge - Accurate paired shotgun read merging via overlap. *PLoS One* 12(10)**,** e0185056. doi: 10.1371/journal.pone.0185056.

Doncheva, N.T., Assenov, Y., Domingues, F.S., and Albrecht, M. (2012). Topological analysis and interactive visualization of biological networks and protein structures. *Nat Protoc* 7(4)**,** 670-685. doi: 10.1038/nprot.2012.004.

GTEx Portal. Data from: *GTEx Analysis V7.* <https://www.gtexportal.org/home/>. (2017).

Hinrichs, A.S., Raney, B.J., Speir, M.L., Rhead, B., Casper, J., Karolchik, D., et al. (2016). UCSC Data Integrator and Variant Annotation Integrator. *Bioinformatics* 32(9)**,** 1430-1432. doi: 10.1093/bioinformatics/btv766.

Kent, W.J., Sugnet, C.W., Furey, T.S., Roskin, K.M., Pringle, T.H., Zahler, A.M., et al. (2002). The human genome browser at UCSC. *Genome Res* 12(6)**,** 996-1006. doi: 10.1101/gr.229102.

Love, M.I., Huber, W., and Anders, S. (2014). Moderated estimation of fold change and dispersion for RNA-seq data with DESeq2. *Genome Biol* 15(12)**,** 550. doi: 10.1186/s13059-014-0550-8.

Mi, H., Muruganujan, A., Ebert, D., Huang, X., and Thomas, P.D. (2019). PANTHER version 14: more genomes, a new PANTHER GO-slim and improvements in enrichment analysis tools. *Nucleic Acids Res* 47(D1)**,** D419-D426. doi: 10.1093/nar/gky1038.

Morrow, J.D., Chase, R.P., Parker, M.M., Glass, K., Seo, M., Divo, M., et al. (2019). RNA-sequencing across three matched tissues reveals shared and tissue-specific gene expression and pathway signatures of COPD. *Respir Res* 20(1)**,** 65. doi: 10.1186/s12931-019-1032-z.

Neale, B. Data from: *UK Biobank GWAS Round 2.* <http://www.nealelab.is/uk-biobank/>. (2018).

Patro, R., Duggal, G., Love, M.I., Irizarry, R.A., and Kingsford, C. (2017). Salmon provides fast and bias-aware quantification of transcript expression. *Nat Methods* 14(4)**,** 417-419. doi: 10.1038/nmeth.4197.

Pons, P., and Latapy, M. (Year). "Computing Communities in Large Networks Using Random Walks": Springer Berlin Heidelberg), 284-293.

Ramakrishnan, V.R., Gonzalez, J.R., Cooper, S.E., Barham, H.P., Anderson, C.B., Larson, E.D., et al. (2017). RNA sequencing and pathway analysis identify tumor necrosis factor alpha driven small proline-rich protein dysregulation in chronic rhinosinusitis. *Am J Rhinol Allergy* 31(5)**,** 283-288. doi: 10.2500/ajra.2017.31.4457.

Shannon, P., Markiel, A., Ozier, O., Baliga, N.S., Wang, J.T., Ramage, D., et al. (2003). Cytoscape: a software environment for integrated models of biomolecular interaction networks. *Genome Res* 13(11)**,** 2498-2504. doi: 10.1101/gr.1239303.

Soneson, C., Love, M.I., and Robinson, M.D. (2015). Differential analyses for RNA-seq: transcript-level estimates improve gene-level inferences. *F1000Res* 4**,** 1521. doi: 10.12688/f1000research.7563.2.

Xia, J., Benner, M.J., and Hancock, R.E. (2014). NetworkAnalyst--integrative approaches for protein-protein interaction network analysis and visual exploration. *Nucleic Acids Res* 42(Web Server issue)**,** W167-174. doi: 10.1093/nar/gku443.

Xia, J., Gill, E.E., and Hancock, R.E. (2015). NetworkAnalyst for statistical, visual and network-based meta-analysis of gene expression data. *Nat Protoc* 10(6)**,** 823-844. doi: 10.1038/nprot.2015.052.

Zerbino, D.R., Achuthan, P., Akanni, W., Amode, M.R., Barrell, D., Bhai, J., et al. (2018). Ensembl 2018. *Nucleic Acids Res* 46(D1)**,** D754-D761. doi: 10.1093/nar/gkx1098.

Zhou, G., Soufan, O., Ewald, J., Hancock, R.E.W., Basu, N., and Xia, J. (2019). NetworkAnalyst 3.0: a visual analytics platform for comprehensive gene expression profiling and meta-analysis. *Nucleic Acids Res*. doi: 10.1093/nar/gkz240.
